# Targeting triple-negative breast cancers with the Smac-mimetic birinapant

**DOI:** 10.1038/s41418-020-0541-0

**Published:** 2020-04-27

**Authors:** Najoua Lalaoui, Delphine Merino, Goknur Giner, François Vaillant, Diep Chau, Lin Liu, Tobias Kratina, Bhupinder Pal, James R. Whittle, Nima Etemadi, Jean Berthelet, Julius Gräsel, Cathrine Hall, Matthew E. Ritchie, Matthias Ernst, Gordon K. Smyth, David L. Vaux, Jane E. Visvader, Geoffrey J. Lindeman, John Silke

**Affiliations:** 1 The Walter and Eliza Hall Institute of Medical Research, Parkville, VIC 3052 Australia; 2 Department of Medical Biology, University of Melbourne, Parkville, VIC 3010 Australia; 3 Olivia Newton-John Cancer Research Institute and School for Cancer Medicine La Trobe University, Heidelberg, VIC 3084 Australia; 4 School of Mathematics and Statistics, University of Melbourne, Parkville, VIC 3010 Australia; 5 Department of Medical Oncology, Peter MacCallum Cancer Centre, Melbourne, VIC 3000 Australia; 6 Department of Medicine, Royal Melbourne Hospital, University of Melbourne, Parkville, VIC 3010 Australia

**Keywords:** Cancer, Cell biology, Molecular biology, Preclinical research

## Abstract

Smac mimetics target inhibitor of apoptosis (IAP) proteins, thereby suppressing their function to facilitate tumor cell death. Here we have evaluated the efficacy of the preclinical Smac-mimetic compound A and the clinical lead birinapant on breast cancer cells. Both exhibited potent in vitro activity in triple-negative breast cancer (TNBC) cells, including those from patient-derived xenograft (PDX) models. Birinapant was further studied using in vivo PDX models of TNBC and estrogen receptor-positive (ER^+^) breast cancer. Birinapant exhibited single agent activity in all TNBC PDX models and augmented response to docetaxel, the latter through induction of TNF. Transcriptomic analysis of TCGA datasets revealed that genes encoding mediators of Smac-mimetic-induced cell death were expressed at higher levels in TNBC compared with ER^+^ breast cancer, resulting in a molecular signature associated with responsiveness to Smac mimetics. In addition, the cell death complex was preferentially formed in TNBCs versus ER^+^ cells in response to Smac mimetics. Taken together, our findings provide a rationale for prospectively selecting patients whose breast tumors contain a competent death receptor signaling pathway for the further evaluation of birinapant in the clinic.

## Introduction

Breast cancer can be categorized into at least five subtypes based on their gene expression profiles [1–5]. Although molecular profiling has contributed to a greater understanding of breast tumor biology, therapeutic approaches are still largely guided by the presence or absence of three biomarkers: estrogen receptor (ER), progesterone receptor (PR), and human epidermal growth factor receptor 2 (HER2) amplification, which broadly divide breast cancer into three groups: luminal (ER^+^ and PR^+^ or PR^–^), HER2-amplified, and triple-negative breast cancer (TNBC), which lacks ER, PR, and HER2 expression [6]. The identification of these biomarkers has assisted the development of targeted therapies that include endocrine therapy (such as tamoxifen or aromatase inhibitors) or anti-HER2 therapy (such as trastuzumab), which have considerably improved survival for women with early and relapsed breast cancer [7, 8]. In contrast, treatment for TNBC still presents a major clinical challenge due to the paucity of useful molecular targets. Apart from patients harboring *BRCA1/2* mutations, where poly (ADP-ribose) polymerase (PARP) inhibitors have recently shown benefit, therapeutic options for patients with TNBC are largely restricted to surgery plus conventional systemic cytotoxic chemotherapy. Recently, Pprogrammed death-ligand 1 (PD-L1) expression has been shown to be a useful biomarker of response to immunotherapy [9]. Therefore, the identification of additional molecular targets for TNBC patients remains an important area of unmet need.

Smac mimetics have emerged as a promising class of targeted therapies that are currently being tested in the clinic for solid and hematological cancers [10]. These drugs suppress the function of inhibitor of apoptosis (IAP) proteins, whose expression is associated with tumorigenesis, chemoresistance, disease progression, and poor prognosis [11]. The key IAP members—XIAP, cIAP1, and cIAP2—bear three tandem baculoviral IAP repeat domains (BIRs) and a C-terminal E3 ligase RING domain. Smac/DIABLO is a natural IAP antagonist protein that, when released from the mitochondria during apoptosis, binds to IAPs [12, 13]. Smac-mimetic compounds are modeled on the N-terminal AVPI tetrapeptide of Smac/DIABLO, which binds to the BIR domains of IAPs, and mimic the inhibitory activity of endogenous Smac/DIABLO.

The development of Smac-mimetic compounds has helped to reveal the role for IAPs in regulating TNF receptor signaling. Binding of TNF to its receptor TNFR1 recruits proteins including cIAP1/2, which conjugate ubiquitin chains to RIPK1, culminating in the activation of NF-κB and MAPK signaling pathways [10]. Inhibition of cIAP1/2 by Smac mimetics reduces the ubiquitylation of RIPK1 and the expression of survival genes such as *CFLAR* encoding cFLIP [10]. Consequently, deubiquitylated RIPK1 binds to caspase-8 to form a deadly cytosolic molecular platform called complex II. Caspase-8 is activated within this complex and cleaves RIPK1 and caspase-3 to induce apoptosis. In some circumstances, caspase-8 activity is insufficient to prevent the formation of a secondary complex consisting of uncleaved RIPK1 and RIPK3, which phosphorylates and activates MLKL to induce necroptosis [14]. By preventing cIAP1/TRAF2/TRAF3-mediated degradation of NF-κB-inducing kinase (NIK), Smac mimetics also induce autocrine secretion of TNF [15–17], and this can further promote cell death in response to Smac mimetics. In contrast to cIAP1/2, XIAP exhibits its antiapoptotic activity through direct binding to and inhibition of caspase-3, -7, and -9 [18]. The binding of Smac mimetics to XIAP’s BIR domains prevents XIAP from directly binding to and inhibiting caspases-3, -7, and -9.

To date, several Smac-mimetic compounds are undergoing investigation in early-phase clinical trials across a range of malignancies including breast cancer [19]. The Smac-mimetic LCL161 (Novartis) has been studied in combination with paclitaxel in a randomized phase II neoadjuvant study in TNBC, where higher pathological Clinical Responses were observed in patients with a TNFα gene expression signature (GS), albeit with significant toxicity. This signature, derived through in silico analysis of LCL161-sensitive versus refractory cell lines, features high *TNF* and *RIPK1* expression and is present in 26% of TNBC [20]. However, it is unknown whether other Smac mimetics like birinapant (TetraLogic Pharmaceuticals/Medivir) elicit similar tumor responses or toxicity. In addition, the molecular mechanisms underlying responsiveness remain to be fully elucidated.

Smac mimetics appear efficacious in some breast cancer cell lines when administrated alone or in combination with death ligands or chemotherapeutic drugs [21]. Breast cancer cell lines exhibit different responses to Smac mimetics [21], suggesting that expression profiles and/or breast cancer subtypes might determine Smac-mimetic responsiveness. In this study, we sought to determine the molecular mechanisms underlying the responsiveness of two breast cancer subtypes to birinapant. We found that birinapant was more potent in TNBC compared with ER^+^ breast cancer cells using primary patient-derived xenografts (PDX) in vitro. Importantly, birinapant was also efficacious in vivo in TNBC PDX models. Protein expression profiling revealed that cIAP1 levels were higher in ER^+^ than TNBC. However, this subtype remained largely resistant to induced cell death, even though birinapant was able to degrade most of cIAP1 in ER^+^ breast cancer cells. Gene expression profiling further revealed that TNBCs express higher levels of *TNF* and other genes encoding activators of Smac-mimetic-induced cell death. Together, our findings reveal a targetable subset of breast cancers that include TNBC and possibly some ER^+^ breast cancers.

## Results

### TNBC PDX tumors are responsive to the Smac-mimetic birinapant

**Fig. 1:**
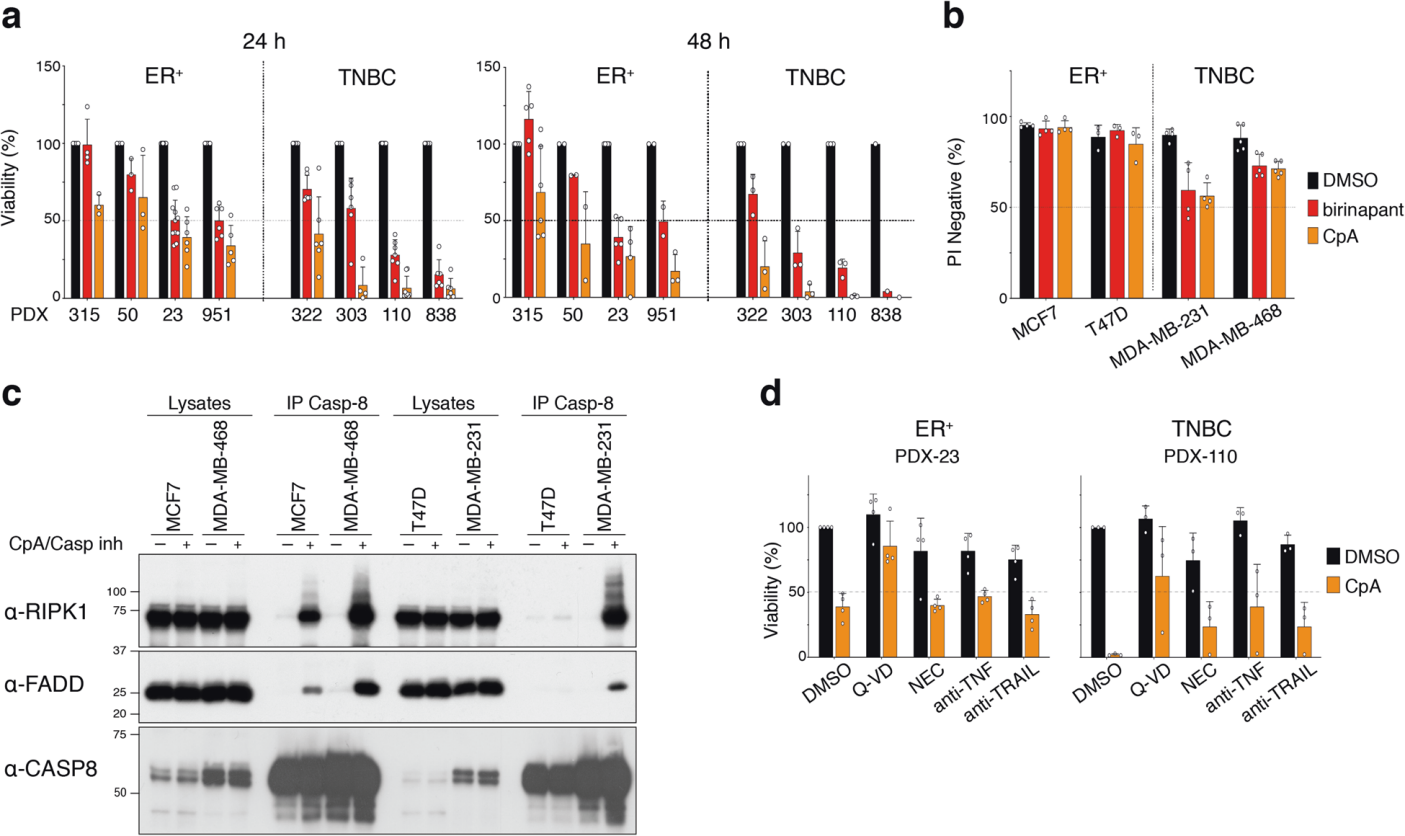
TNBC are highly sensitive to Smac mimetics. **a** Cell viability using CellTiter-Glo of the indicated ER^+^ and TNBC PDX tumor cells treated for 24 or 48 h with 1 μM of birinapant or CompA (CpA) in mammosphere medium. **b** Cell viability assessed by measurement of Propidium iodide (PI) negative cells by flow cytometry of indicated breast cancer cell lines treated for 24 h with 1 μM of birinapant or CompA (CpA). **c** Western blot analysis of complex II/Ripoptosome using anti-caspase-8 antibody. Cells were treated for 5 h with 1 μM of CompA (CpA) and with 5 μM of the caspase inhibitor IDN-6556 (Casp inh) to stabilize the complex. **d** Cell viability assessed using CellTiter-Glo of the ER^+^ PDX-23 and the TNBC PDX-110 tumor cells treated for 24 h with 1 μM CompA (CpA) ±10 μM of Q-VD-OPh (Q-VD) or 50 μM Necrostatin (NEC) or 10 μg/ml of blocking anti-TNF or 10 μg/ml of blocking anti-TRAIL. **a**, **b**, **d** Results are presented as percentages of untreated cells. Bars are means ± SD; *n* = 1–6 independent tumors per PDX and *n* = 3–5 independent experiments for cell lines. Each dot represents an independent tumor or an independent experiment.

To test the sensitivity of breast cancers to IAP inhibition, we evaluated the potency of two bivalent Smac mimetics on primary breast cancer tumors. Using a short-term tumor sphere assay [22], we tested the preclinical pan-IAP Smac-mimetic Compound A (CpA) and the clinical lead compound birinapant on a panel of ER^+^ and TNBC PDX models. All tumors were sensitive to CpA-induced killing over 24 h, most notably the TNBC models (Fig. 1a). Although birinapant is a less potent inhibitor of XIAP and cIAP2 than CompA [23], PDX cells were also sensitive to this drug. In most cases, Smac-mimetic-mediated cell death requires the presence and/or secretion of autocrine TNF or another death ligand [15–17, 24–26]. Therefore, we determined cell viability at 48 h to provide ample time for an autocrine ligand to be secreted and mediate cell killing. At this time-point, treatment produced substantial cell death in TNBC PDX tumor cells (Fig. 1a). The TNBC cell lines MDA-MB-231 and MDA-MB-468 also exhibited sensitivity to Smac mimetics, while ER^+^ MCF-7 and T47D breast cancer cells appeared refractory (Fig. 1b). Consistent with these findings, formation of the cell death complex was limited in ER^+^ compared with TNBC cell lines (Fig. 1c).

Treatment with a Smac-mimetic can cause either caspase-dependent apoptosis or trigger RIPK1/RIPK3-dependent necroptosis [27]. To explore the mechanism underpinning Smac-mimetic-induced cell death in PDX-derived spheres, we used the pan-caspase inhibitor, Q-VD-OPh and the RIPK1 inhibitor, necrostatin. Inhibition of RIPK1 partially protected against Smac-mimetic-induced cell death in PDX-110, whereas Q-VD-OPh substantially blocked cell death induced by the Smac-mimetic in both PDX models (Fig. 1d). These findings suggest that Smac mimetics primarily kill breast cancer cells by caspase-dependent apoptosis. Consistent with the known mechanism of action of Smac mimetics, both TNF and TRAIL neutralizing antibodies reduced Smac-mimetic killing in the TNBC PDX-110, suggesting that both autocrine TNF and TRAIL contribute to Smac-mimetic-induced killing (Fig. 1d).

**Fig. 2:**
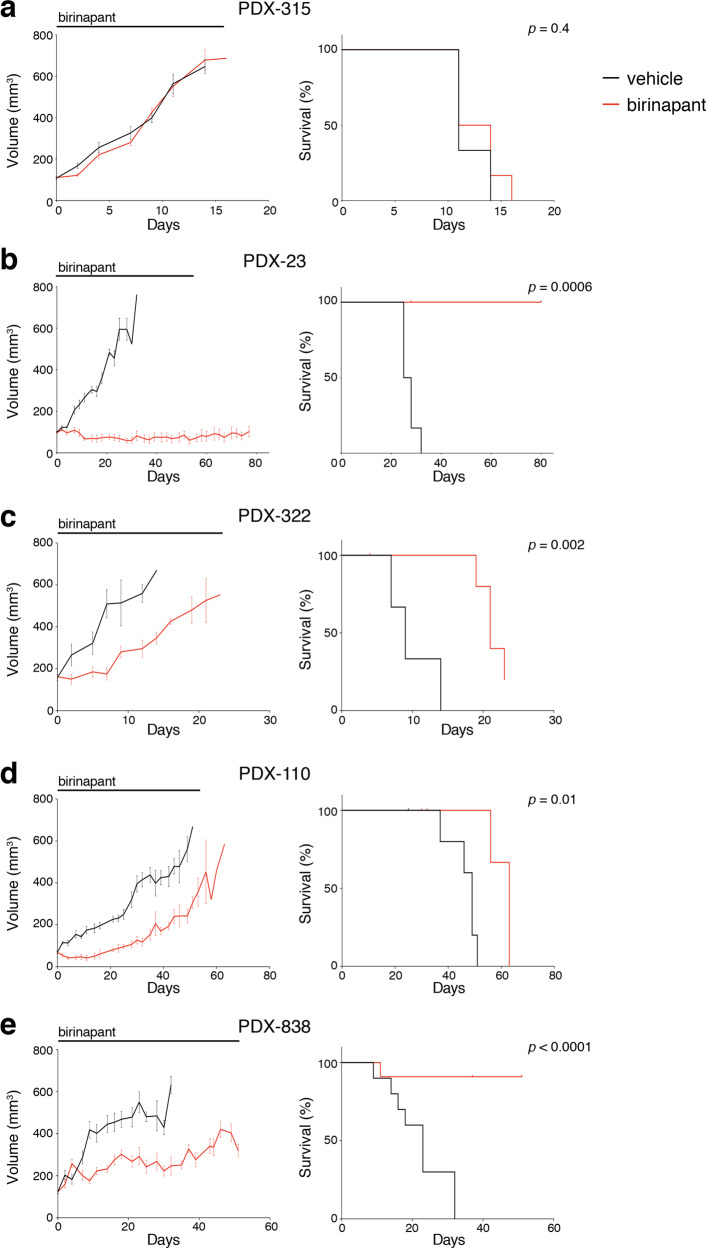
In vivo efficacy of birinapant in PDX models. **a**–**e** Tumor volume curves (left panels) and Kaplan–Meier survival curves (right panels) for the indicated PDX model (*n* = 6 to 10 mice per arm). Mice were treated with vehicle alone (black lines) or 30 mg/kg of birinapant (red lines) intraperitoneally three times/week. The treatment time is represented by the gray bars on top of the tumor volume curves. Mice were sacrificed when tumor size reached the experimental ethical end point (>600 mm^3^). For tumor volume curves, means ± SEM are shown.

We next evaluated the in vivo response to the clinical compound birinapant in PDX models, including two ER^+^ models and three TNBC models. Mice were treated with birinapant (30 mg/kg i.p.) three times per week for up to seven weeks. Therapy was well-tolerated, with mice maintaining normal body weight during therapy (not shown), and no perturbation of full blood count was observed following birinapant therapy (Supplementary Fig. 1). Similar to responses observed in vitro, birinapant did not impact on tumor growth of the PDX-315 ER^+^ model but significantly attenuated the growth of PDX-23 and all three TNBC models (PDX-110, -838, and -322), accompanied by significant improvement in survival to ethical endpoint (Fig. 2 and Supplementary Fig. 2). Together these data suggest that TNBCs are more sensitive to killing by Smac mimetics than ER^+^ tumors.

### cIAP1 is differentially expressed between ER^+^ and TNBC tumors

**Fig. 3:**
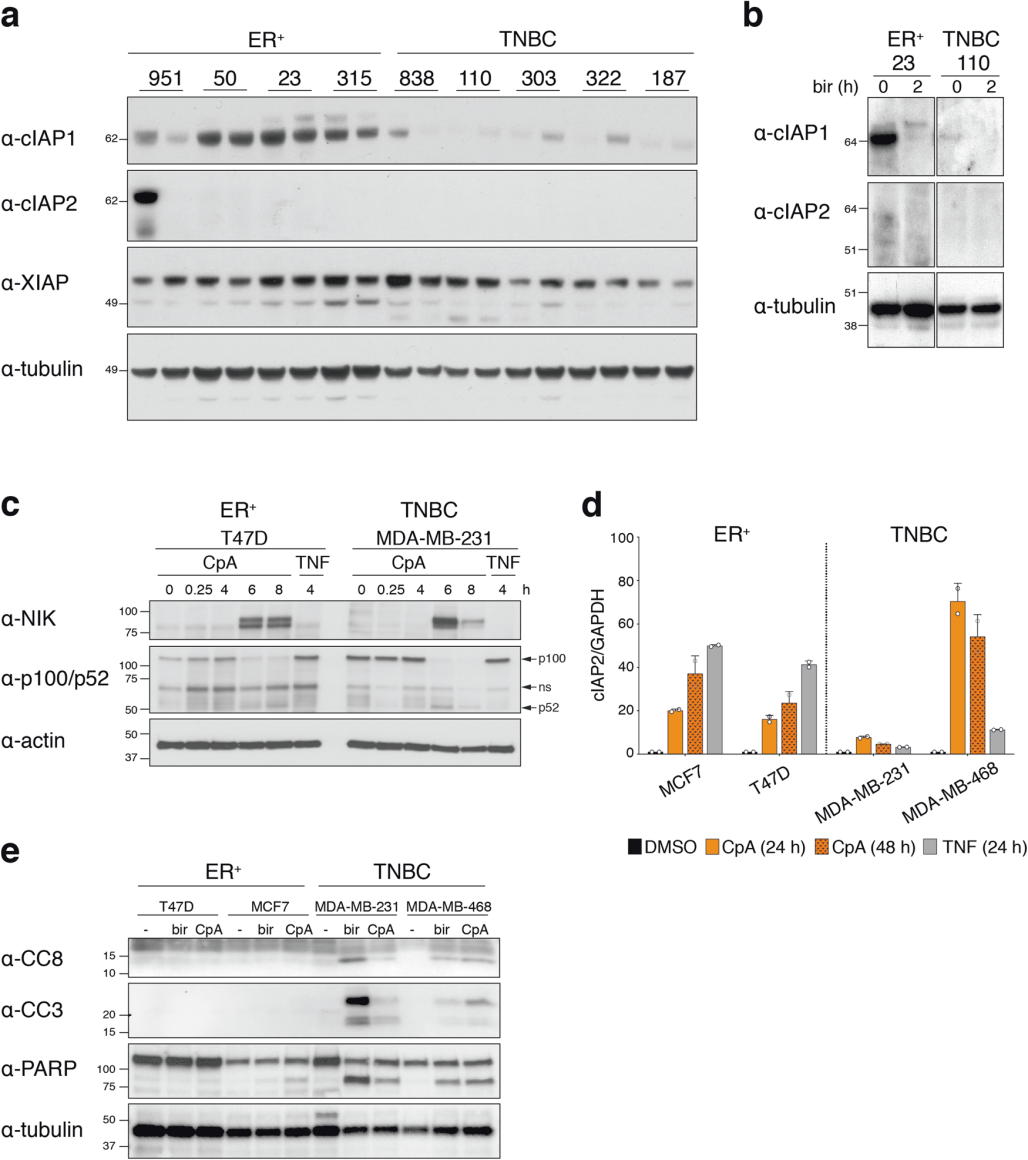
Differential expression of cIAP1/2 in ER^+^ and TNBC tumors. **a** Western blot analysis of cIAP1, cIAP2, and XIAP protein expression in the indicated ER^+^ and TNBC PDX models. For each PDX, two lysates from independent mice are represented. **b** Western blot analysis of cIAP1 and cIAP2 protein expression in lysates from ER^+^ PDX-23 and TNBC PDX-110 cell suspensions treated with 1 μM of birinapant for 2 h. **c** Western blot analysis of NIK, p100/p52 protein expression in indicated breast cancer cell lines treated at indicated times with 1 μM of CompA (CpA) or with 100 ng/ml of TNF. **d** Levels of cIAP2 transcripts relative to GAPDH transcripts in indicated breast cancer cell lines treated with 1 μM of CompA (CpA) for 24 h or 48 h or with 100 ng/ml of TNF for 24 h. The caspase inhibitor IDN-6556 (5 μM) was added in all conditions to inhibit cell death. Bars represent mean ± SD of duplicates of one representative experiment. **e** Western blot analysis of cleaved caspase-3 (CC3), cleaved caspase-8 (CC8) and PARP  in lysates from indicated breast cancer cell lines treated with 1 μM of birinapant (bir) or CompA (CpA) for 24 h. Tubulin (**a**, **b**) and actin (**c**, **e**) were used as loading controls.

Smac mimetics bind to the BIR domains of IAPs and thereby inhibit their function. In the case of cIAP, Smac mimetics induce auto-ubiquitylation and consequent degradation by the proteasome [15, 17]. Because CompA and birinapant are structurally similar, but have different affinities to the IAPs, we evaluated expression of their targets cIAP1, cIAP2, and XIAP in ER^+^ and TNBC PDX tumors to understand the differential responsiveness of the breast cancer subtypes to Smac mimetics. There were no major differences in XIAP levels between ER^+^ and TNBC PDX tumors and cIAP2 was undetectable in most tumors (Fig. 3a). This is consistent with the observation that cIAP2 is a gene induced in response to NF-κB activation [28, 29]. Notably, however, much lower levels of cIAP1 were observed in TNBC PDX tumors compared with ER^+^ PDX models (Fig. 3a). It is possible that the higher cIAP1 level in ER^+^ tumors accounted for their relative resistance to Smac mimetics. Despite the high level of cIAP1 in PDX-23, birinapant effectively induced cIAP1 degradation within 2 h (Fig. 3b).

cIAP1/2 are known to promote activation of canonical NF-κB signaling in response to TNF, while inhibiting spontaneous activation of the noncanonical NF-κB signaling pathway by constitutively degrading the upstream activating kinase, NIK [15, 17, 30]. Thus, inhibition of cIAP1/2 by Smac mimetics results in the processing of NF-κB2 p100 into p52, causing transcription of NF-κB dependent genes, including cIAP2. Consistent with this, treatment of both ER^+^ and TNBC cell lines with the Smac-mimetic CpA-induced stabilization of NIK and consequent processing of p100 into p52 (Fig. 3c). Although NIK accumulation was higher in TNBC cells, the magnitude of p100 processing was similar in both ER^+^ and TNBC cell lines (Fig. 3c). Accordingly, we found that levels of *cIAP2* transcripts increased after Smac-mimetic treatment in both ER^+^ and TNBC cell lines (Fig. 3d). Despite the ability of Smac mimetics to activate the noncanonical NF-κB pathway in ER^+^ breast cancer subtypes, both birinapant and CpA were more effective in activating caspases and inducing cell death in TNBC cell lines, consistent with formation of the larger complex II in TNBC cells (Figs. 1a–c and 3e).

### A gene expression signature in TNBC containing mediators of Smac-mimetic killing

**Fig. 4:**
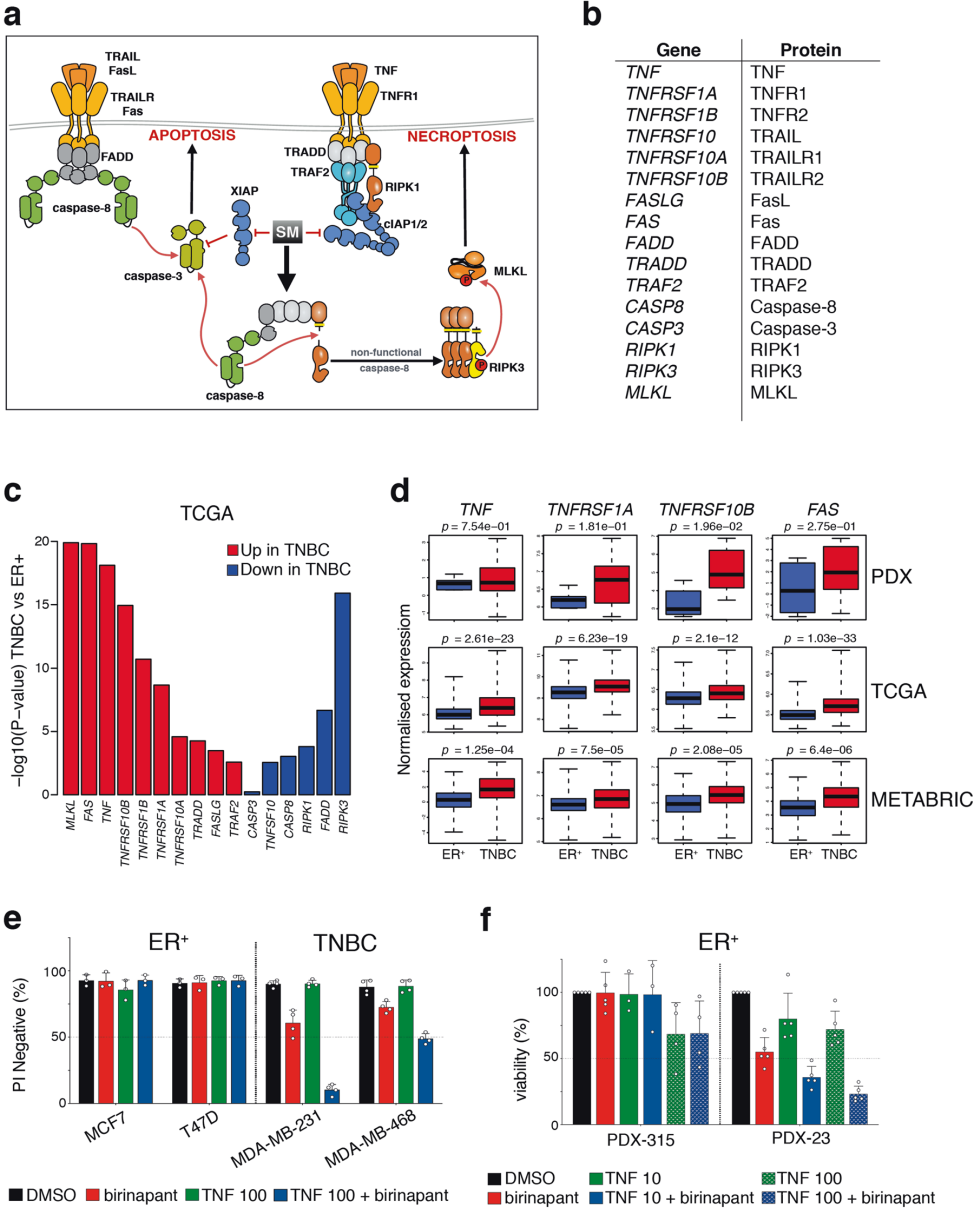
A gene expression profile that correlates with Smac-mimetic killing of TNBC. **a** Schematic of death receptor cell death signaling pathway. Effect of Smac-mimetic (SM)-induced inhibition of IAPs (red inhibitory arrows) and -induced formation of cell death complex (black arrow) are shown. **b** List of selected genes and their protein names that influence Smac-mimetic-induced cell death. **c** Most Smac-mimetic killing genes are upregulated in TCGA TNBC samples compared with ER^+^ cancers (*n* = 132 for ER^+^ and *n* = 183 for TNBC, *p* = 0.0005) but a minority are downregulated. The plot shows −log10 *p* value and the direction of change for each gene in TNBC versus ER^+^ samples. **d** Box plots representing the expression of indicated genes in PDX models (top panels, *n* = 3 for ER^+^ and *n* = 7 for TNBC PDX models), in TCGA samples (middle panels, *n* = 132 for ER^+^ and *n* = 183 for TNBC samples) and in METABRIC samples (bottom panels, *n* = 492 for ER^+^ and *n* = 331 for TNBC samples). **e** Cell viability assessed by measurement of PI negative cells by flow cytometry of indicated breast cancer cell lines treated for 24 h with 1 μM of birinapant or 100 ng/ml of TNF or combination of both. Data are means ± SD; *n* = 2–3 independent experiments. **f** Cell viability assessed using CellTiter-Glo of ER^+^ PDX tumor cells treated for 24 h with 1 μM of birinapant or 10 ng/ml or 100 ng/ml of TNF (indicated as TNF 10 or TNF 100, respectively) or combination of birinapant with either TNF concentrations. Data are means ± SD; *n* = 3–5 independent tumors. **e**, **f** Each dot represents either an independent tumor or an independent experiment.

Smac mimetics function by inhibiting IAPs but also require the expression of other downstream death effector proteins in order to kill cancer cells [26]. Using The Cancer Genome Atlas (TCGA) gene dataset (CGAN, 2012), we compared the expression of genes involved in cell death pathways between ER^+^ and TNBCs. Interestingly, genes linked to the gene ontology (GO) term ‘programmed cell death’ (‘PCD’) were generally found to be expressed at higher levels in TNBC versus ER^+^ samples (FRY gene set test *p* = 0.0005). As the ‘PCD’ GO does not solely include genes that are involved in cell death induced by Smac mimetics, we refined our analysis to a subset of genes that we considered more likely to contribute to Smac-mimetic killing (Fig. 4a, b). Using this refined ‘Smac-mimetic’ signature, most genes encoding death receptor family members were strongly upregulated in patients with TNBC compared with those with ER^+^ cancer (Fig. 3c and Supplementary Fig. 3). Significance for overall upregulation is *p* = 5.6e−06 compared with all PCD genes despite the smaller gene set size. Interestingly, *RIPK1* and other genes encoding components of complex II (or Ripoptosome) were found to be downregulated in TNBC (Fig. 4c).

Given that treatment with birinapant removed differences in IAP levels between TNBC and ER^+^ tumors (Fig. 3b), we postulated that differential levels of TNF and TNFR1 could account for ER^+^ resistance and TNBC sensitivity to Smac mimetics. Consistent with this notion, transcripts encoding members of the death receptor family such as TNF (*TNF*), TNFR1 (*TNFRSF1*), FAS (*FAS*), or TRAIL-R2 (*TNFRSF10B*) were significantly higher in the TCGA TNBCs compared with ER^+^ tumors (Fig. 4c, d). This differential expression was also observed in an independent dataset (METABRIC) covering 2000 breast cancers [31] as well as in our cohort of 10 PDX tumors (Fig. 4d). Furthermore, a neutralizing TNF antibody blocked Smac-mimetic killing of TNBC PDXs, while addition of exogenous TNF further sensitized TNBC cells to birinapant (Figs. 1d, 4e, f). In contrast, addition of TNF did not increase complex II formation in ER^+^ cells (Supplementary Fig. 4), nor enhance birinapant-mediated killing in ER^+^ cell lines or the PDX-315 ER^+^ model, although TNF slightly increases birinapant-mediated killing in PDX-23 (Fig. 4e, f). These results suggest that the basal levels of TNFR1 in ER^+^ tumors are generally not high enough for Smac mimetics to initiate cell death.

### Birinapant sensitizes TNBC PDX tumors to taxane therapy in vivo

**Fig. 5:**
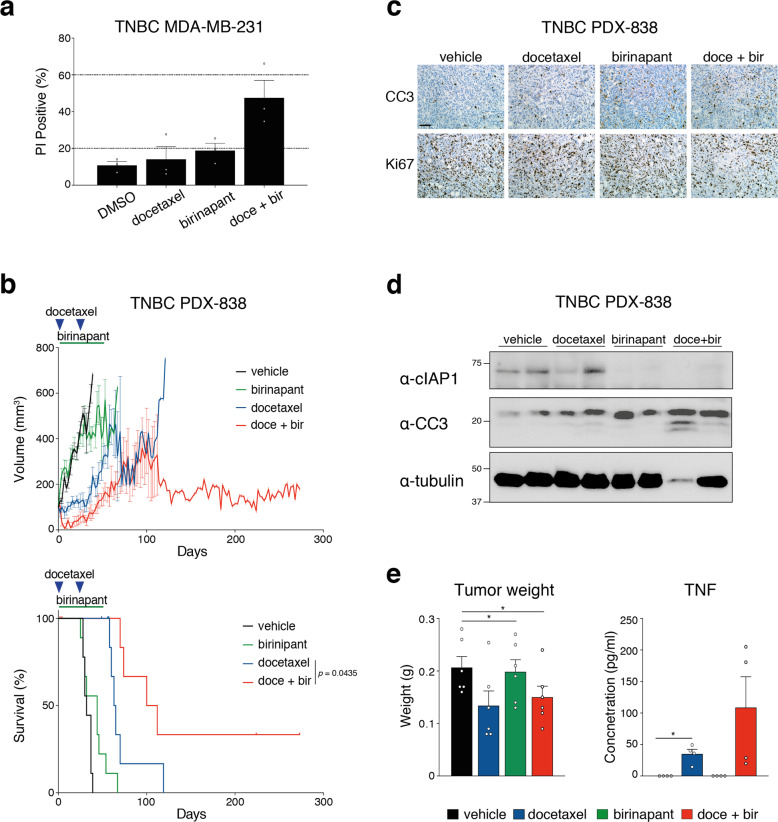
Birinapant sensitizes TNBC PDX tumors to conventional therapy in vivo. **a** Cell death assessed by measurement of PI positive cells by flow cytometry of MDA-MD-231 cells treated with either 5 nM of docetaxel for 48 h or 250 nM of birinapant for 24 h or pretreated 24 h with 5 nM of docetaxel and then treated 24 h with 250 nM birinapant (doce + bir). Data are means ± SEM; *n* = 3 independent experiments. **b** Tumor volume curves (top panels) and Kaplan–Meier survival curves (bottom panels) for TNBC PDX-838 (*n* = 6–9 mice per arm). Mice were treated with vehicle alone (black line) or 15 mg/kg of birinapant alone (green line, intraperitoneally three times/week for seven weeks) or with 10 mg/kg docetaxel alone (blue line, intraperitoneally on days 1 and 22) or with combined docetaxel and birinapant (red line). Mice were sacrificed when tumor size reached the experimental ethical end point (>600 mm^3^). For tumor volume curves, means ± SEM are shown. **c** Immunostaining for cleaved caspase-3 (CC3) and Ki67 of PDX-838 tumors treated in vivo with vehicle or with 15 mg/kg of birinapant or with 10 mg/kg docetaxel or with combined docetaxel and birinapant for 24 h. Scale bar, 50 μm. **d** Western blot analysis of cIAP1 and cleaved caspase-3 protein expression in lysates from PDX 838 tumors treated in vivo with vehicle or with 15 mg/kg of birinapant or with 10 mg/kg docetaxel or with combined docetaxel and birinapant for 24 h (two independent tumors per treatment). **e** Tumor weights from PDX 838 tumors treated in vivo with vehicle or with 15 mg/kg of birinapant or with 10 mg/kg docetaxel or with combined docetaxel and birinapant for 24 h (left panel). Level of TNF measured by ELISA in lysate from PDX-838 tumors treated in vivo with vehicle or 15 mg/kg of birinapant or with 10mg/kg docetaxel or with combined docetaxel and birinapant for 24 h (right panel). Data are means ± SD; *n* = 4–6 independent tumors. Each dot represents an independent tumor.

Although birinapant was effective as a single agent in TNBC PDX models in vivo, combination therapy could prove more potent. We therefore tested the ability of birinapant to sensitize TNBCs to the taxane docetaxel, which is widely used in breast cancer therapy. Birinapant enhanced killing by docetaxel in MDA-MB-231 cells in vitro (Fig. 5a). We next determined the therapeutic effect of this combination in the PDX-838 model of TNBC in vivo. Mice were treated with docetaxel (10 mg/kg i.p.) every 21 days for two cycles with or without birinapant (15 mg/kg i.p.) administered three times a week for seven weeks (Fig. 5b and Supplementary Fig. 5). At this dose, birinapant alone was insufficient to inhibit tumor growth. When combined with docetaxel, however, tumor growth was significantly curtailed, accompanied by a significant improvement in animal survival (median survival 66 days versus 106 days; *p* < 0.05) (Fig. 5b). As expected, combined therapy increased apoptosis and reduced tumor cell proliferation, as determined by immunostaining for cleaved caspase-3 (CC3) and Ki67 (Fig. 5c) and Western blot analysis of CC3 (Fig. 5d). It has been reported that docetaxel can stimulate TNF production in breast cancer cells [32, 33]. To determine whether TNF induced by docetaxel synergized with birinapant, we measured TNF levels in tumor lysates and in the serum of treated mice. Docetaxel or docetaxel plus birinapant significantly reduced tumor weight at 24 h (Fig. 5e, left panel), accompanied by an increase in TNF expression by docetaxel and further elevation by its combination with birinapant (Fig. 5e, right panel). These findings are consistent with docetaxel sensitizing TNBC to birinapant via the induction of TNF.

## Discussion

There remain few targeted therapies available for patients with TNBC, underscoring the importance of identifying new targets. In this study, we have explored the IAP inhibitor birinapant as a possible targeting strategy and found that single agent therapy produces a therapeutic response in TNBC PDX models. Our findings shed light on possible preferential activity of Smac mimetics in TNBC, which is consistent with the elevated expression of genes encoding mediators that could influence Smac-mimetic killing in TNBC compared with ER^+^ breast cancer. This is in keeping with a recent description of a TNFα gene expression signature that is enriched in TNBC [20].

The high level of cIAP1 in ER^+^ PDX and their resistance to Smac-mimetic-induced cell death appears counter-intuitive. Indeed, our assumption had been that cells with the highest levels of IAPs would be the most addicted to IAPs and the most sensitive to inhibition. However, despite rapid cIAP1 degradation and higher gene expression of the main components of complex II, Smac mimetics failed to efficiently activate caspases and kill ER^+^ tumors. We previously showed that ER^+^ tumors undergo apoptosis following BCL-2 inhibition [34], demonstrating that the executioner caspases are functional in this breast cancer subtype. Taken together, our findings suggest that most ER^+^ tumors, in contrast to TNBC, are deficient in their ability to form and/or activate complex II. Recent reports showed that complex II activity can be limited by the kinases IKKα, β, ε, p38, MK2, TBK1, or by the E3 ligase MIB2 [35–42]. These proteins restrain the cytotoxic activity of RIPK1 upon Smac-mimetic treatment [35, 36]. Interestingly, the level of IKKα/β transcripts was significantly lower in TNBC compared with ER^+^ tumors in TCGA samples, which may in part account for the ability of TNBC cells to activate the cytotoxic activity of RIPK1 (Supplementary Fig. 6). In contrast, while *MIB2* expression shows an opposite trend and is elevated in TNBC relative to ER^+^ cancers, we did not find differences in the expression of *MAPK14* (p38) or *MAPKAP2* (MK2) between TCGA ER^+^ and TNBC samples (Supplementary Fig. 6). Because RNA transcript levels do not always predict protein activity, it will be interesting to further explore the role of these ‘RIPK1 blockers’ in ER^+^ breast cancers.

For most Smac mimetics, the level of cIAPs and their degradation upon treatment are readily ascertainable biomarkers of drug-target engagement and drug response [43, 44]. Although cancers may select for high levels of IAPs [45, 46], our data suggest that screening for IAP levels and degradation may not predict responsiveness. One feature of Smac mimetics is that they have a two-pronged mechanism of action: they induce TNF and simultaneously sensitize cells to this ligand. Thus, if cancer cells fail to produce TNF upon Smac-mimetic treatment, exogenous TNF would sensitize them to Smac mimetics only if they express TNFR1. Consistent with this, treatment with birinapant and high concentrations of exogenous TNF did not sensitize ER^+^ cancer cells, presumably reflecting their low levels of TNFR1.

The efficacy of Smac mimetics relies on sufficient quantities of TNF, which can be augmented by other means, including innate immune stimuli [47–49], p38, MK2, or caspase inhibitors [37, 50]. Although high levels of TNF can be a concern in regard to safety, these combinations have been proven to be well-tolerated in mice [37, 47, 49, 50]. The combination of birinapant and docetaxel may also offer a dual mechanism of action to achieve clinical benefit as both agents augment TNF production. The safety of the combination of the Smac-mimetic LCL161 with a taxane has been evaluated in the clinic (NCT01188499). Cytokine release syndrome (CRS) appeared to be a dose limiting toxicity for patients treated with both LCL161 and paclitaxel [20]. Importantly, this adverse event has so far not been reported in patients treated with birinapant alone or when combined with docetaxel (NCT01188499). The highly inflammatory cytokine IL-1β can be released when all IAPs are inhibited [51–55]. Consistent with this idea and due to its ability to efficiently target all IAPs, LCL161 can induce the release of IL-1β in different cell types, while birinapant cannot [23, 52]. Therefore, it is plausible that release of IL-1β participated in the CRS observed in patients treated with LCLl61. Conversely, cytokine induction by Smac mimetics could enhance an anti-tumor immune response [56–58], and combinations with immune checkpoint inhibitors may be worth investigating given recent findings with nab-paclitaxel and the PD-L1 inhibitor atezolizumab in PD-L1 positive TNBC [9].

Our findings are in line with a recently published study that identified a predictive gene signature enriched in TNBC, which also included *TNF* [20]. Our Smac-mimetic gene set revealed increased *TNF* levels but also showed elevated expression of genes encoding death receptors and their ligands in TNBC. These factors, if absent or expressed at low levels, would significantly limit Smac-mimetic efficacy. Collectively, our data suggest that the Smac-mimetic gene set may serve as a predictive biomarker to stratify patients for Smac-mimetic-based therapies and support additional early-phase clinical evaluations of birinapant combined with docetaxel in TNBC patients.

## Materials and methods

### Reagents and antibodies

Birinapant, CompA, necrostatin, and IDN-6556^50^ were gifts from TetraLogic Pharmaceuticals. Fc-TNF was generated in-house. Q-VD-OPh (Q-VD) was purchased from MP Biomedicals. Docetaxel was purchased from ActiveBiochem. Antibodies used for neutralization assays anti-TNF (MAB610) and anti-TRAIL (MAB375) were purchased from R&D Systems. Antibodies used for immunoblotting and immunohistochemistry were purchased as follows: anti-cIAP1 (1E1-1-10) and anti-cIAP2 (16E-6-3) from Enzo, anti-XIAP (2F1) from MBL, anti-cleaved caspase-3 (Asp175) from Cell Signaling Technology), anti-NIK (Cell Signaling Technology), anti-p100/p52 (Cell Signaling Technology), anti-tubulin (DM1A) and anti-actin (AC-15) from Sigma-Aldrich, and anti-KI67 (Thermo Fisher Scientific).

### Viability assays

The breast cancer cell lines MCF-7, T47D, MDA-MB-231, and MDA-MB-468 were maintained in RPMI-1640 (Gibco) supplemented with 10% fetal calf serum (FCS) and were mycoplasma free. For viability assays, breast cancer cell lines were plated at 5 × 10^4^ cells/well in 48 well plates and treated with indicated reagents. Propidium iodide exclusion (5 μg/ml) was analyzed by flow cytometry. For tumor sphere assays, single cell suspensions were obtained by digestion of primary tumors, sorted and cultured in mammosphere medium as previously described [34]. 3 × 10^4^ cells/well were plated in 96 well plates and treated with indicated reagents. Cell viability was assessed using the CellTiter-Glo Luminescent Assay (Promega) as per the manufacturer’s instructions.

### In vivo experiments

Human breast cancer tissues were obtained from consenting patients through the Royal Melbourne Hospital Tissue Bank and the Victorian Cancer Biobank with relevant institutional review board approval and were used to derive the PDX models (characterized in [22, 34, 59]). Human ethics approval was obtained from the Walter and Eliza Hall Institute (WEHI) Human Research Ethics Committee. Animal experiments were approved by the WEHI Animal Ethics Committee. Cohorts of NOD-SCID-IL2Rγ^−/−^ female mice were seeded with thawed single cell suspensions of early passage human breast tumors (passage 2 or 3). Briefly, 150,000–250,000 cells were resuspended in 10 μl of transplantation buffer (50% FCS, 10% of a 0.04% trypan blue solution, and 40% PBS) and growth factor-reduced Matrigel [BD] at a ratio of 3:1, and injected into the cleared mammary fat pads of 3–4 weeks old NOD-SCID-IL2Rγ_c_^−/−^ female mice. Mice were monitored for tumor development three times weekly and tumor size measured using electronic vernier calipers. Tumor volume was estimated by measuring the minimum and maximum tumor diameters using the formula: (minimum diameter)^2^ (maximum diameter)/2. Once tumors reached a volume of 80–120 mm^3^, mice were randomized into treatment arms and treatment commenced. As power analysis was not possible in the absence of prior knowledge on the drug effect, we used the equation as described in http://www.3rs-reduction.co.uk/html/6__power_and_sample_size.html. Based on the equation, all experiments described have an *E* value equal or superior to 10. Randomization and tumor measurements were managed using the Study Director software (v 3.0, studylog). Mice were sacrificed at the first measurement where tumor volume exceeded 600 mm^3^, or if their health deteriorated for reasons other than disease progression or drug toxicity (censored event). Although >10% weight loss was a predefined censoring event, no mice in the treatment cohorts lost weight. Animal technicians and researchers blinded to treatment conditions measured the tumors volume and euthanized the mice on ethical grounds. For single agent treatments vehicle for birinapant (6% captisol) or birinapant (30 mg/kg) were injected i.p. three times weekly continuously. For combination treatments vehicle (6% captisol) or birinapant (30 mg/kg) were injected i.p. three times weekly for seven weeks. Docetaxel (10 mg/kg) was injected i.p. every 21 days for two treatment cycles. For the analysis of CC3, Ki67, and TNF, mice were treated with either vehicle (6% captisol) or birinapant (30 mg/kg) or docetaxel (10 mg/kg) or combined birinapant and docetaxel. After 24 h of treatment, tumors were collected and sectioned in two pieces. One was used for immunohistochemistry (detail see below) and the other for TNF measurement. For the TNF ELISA, tumors were lysed in RIPA buffer (20 mM Tris-HCl pH 7.5, 135 mM NaCl, 1.5 mM MgCl_2_, 1 mM EGTA, 1% Triton X-100, and 10% glycerol). Protein lysates were analyzed by ELISA following the manufacturer’s instructions (eBioscience).

### Immunoblot analysis

Tumors were lysed in RIPA buffer (20 mM Tris-HCl pH 7.5, 135 mM NaCl, 1.5 mMMgCl_2_, 1 mM EGTA, 1% Triton X-100, and 10% glycerol). Protein lysates were analyzed by Western blot on 4–15% SDS-polyacrylamide gels (BioRad), transferred onto PVDF membranes (Millipore), and probed with indicated primary antibodies. After primary antibody, membranes were probed using HRP-conjugated anti-IgG secondary antibodies and ECL (GE Healthcare Life Sciences).

### Immunohistochemistry

PDX tumors were collected and fixed in 10% neutral buffered formalin before embedding in paraffin. Sections were dewaxed and subjected to heat-induced epitope retrieval with boiling citrate buffer, then blocked and permeabilized with 1% bovine serum albumin and 0.3% Triton X-100. Immunohistochemistry sections were stained with anti-CC3 (Cell Signaling Technology) or anti-Ki67 (Thermo Fisher Scientific) at 4 °C overnight and followed by anti-rabbit secondary (Agilent Technologies). Signal detection was performed using ABC Elite (Vector Labs) for 30 min and 3,3′-diaminobenzidine (Dako) for 5 min at room temperature.

### RNA-seq analysis of PDX tumors

RNA from PDX tumors was sequenced on a HiSeq 2000 at the Australian Genome Research Facility, Melbourne. Xenografts were derived from three ER^+^ and seven TNBC patients, and each tumor was passaged in 2–4 mice. An average of 20 million 100 bp paired-end reads were obtained per sample. Reads were aligned to the human genome hg19 using Rsubread package 1.16.1 [60] and were assigned to Entrez genes using featureCounts [61]. Library sizes were normalized by Trimmed Median of *M*-values [62] and converted to log2 counts per million (logCPM) using the edgeR package.

### Publicly available breast cancer datasets

RSEM counts for the TCGA breast cancer tumors were downloaded from https://tcga-data.nci.nih.gov and converted to logCPM values using edgeR. Differential expression between TNBC and ER^+^ tumors was assessed using the limma software package [63] and the voom method [64]. Gene set tests were conducted using the FRY method [65] to determine whether the average log-expression of all genes in the set is changed between tumor types. Normalized microarray expression data from the METABRIC project [31] were downloaded from http://www.compbio.group.cam.ac.uk/publications/supplementary-material.

### Statistical analysis

Unless otherwise specified, data are presented as mean ± SD. For tumor weight and TNF ELISA comparisons were performed with a Student’s *t* test and for Kaplan–Meier survival curves comparisons were performed with a log-rank (Mantel–Cox) test. All *p* values are denoted in the figures.

## Supplementary information

SupFig1

SupFig2

SupFig3

SupFig4

SupFig5

SupFig6

Supplementary Figures and Legends
