# Supplementary figures and images for: Targeting triple-negative breast cancers with the Smac-mimetic birinapant
Source: Cell Death Differ. 2020 Apr 27;27(10):2768–80. doi: 10.1038/s41418-020-0541-0 (PMC7492458; doi:10.1038/s41418-020-0541-0)

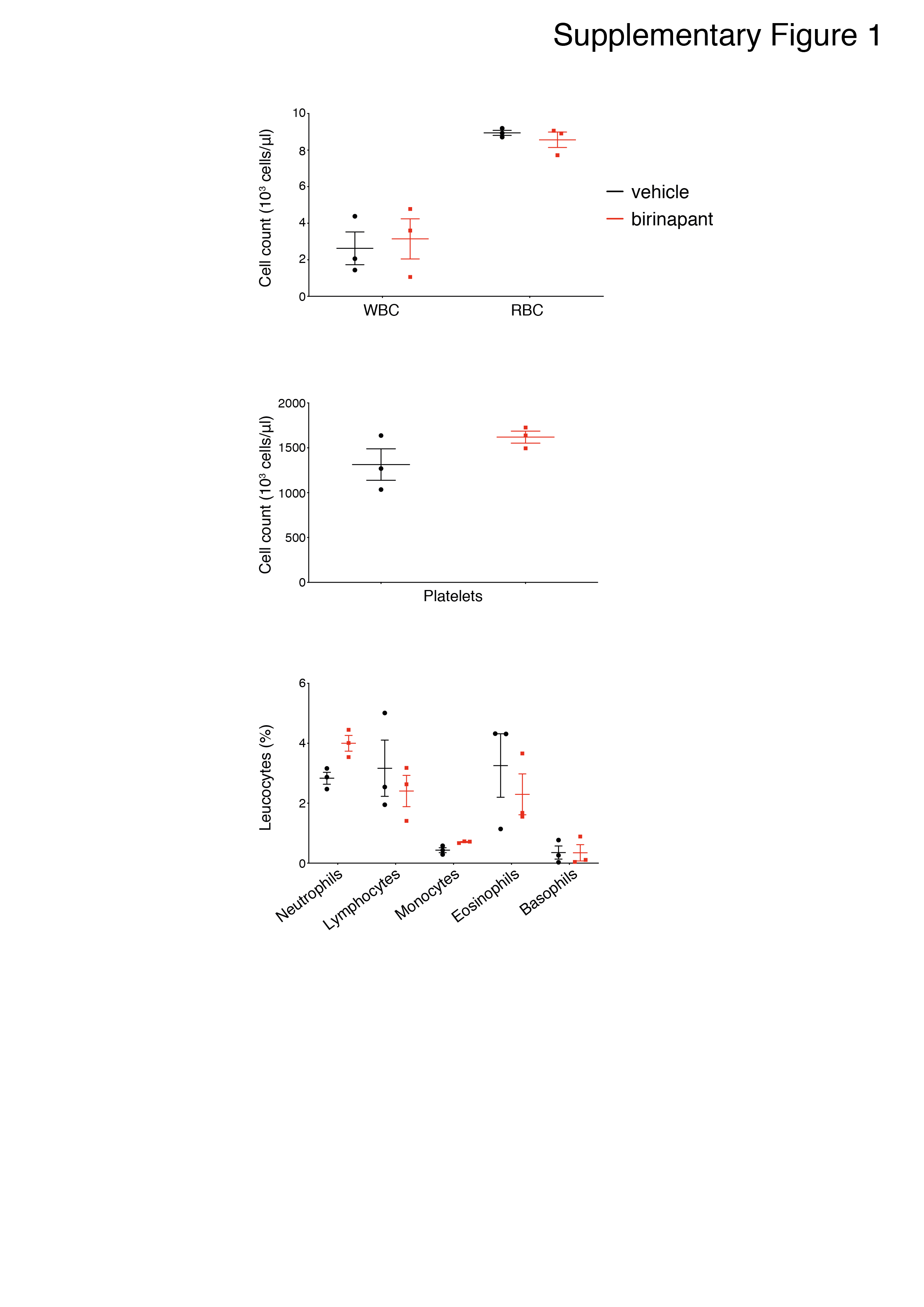

Supplement: Supplementary file 1 — SupFig1 [file 41418_2020_541_MOESM1_ESM.png]

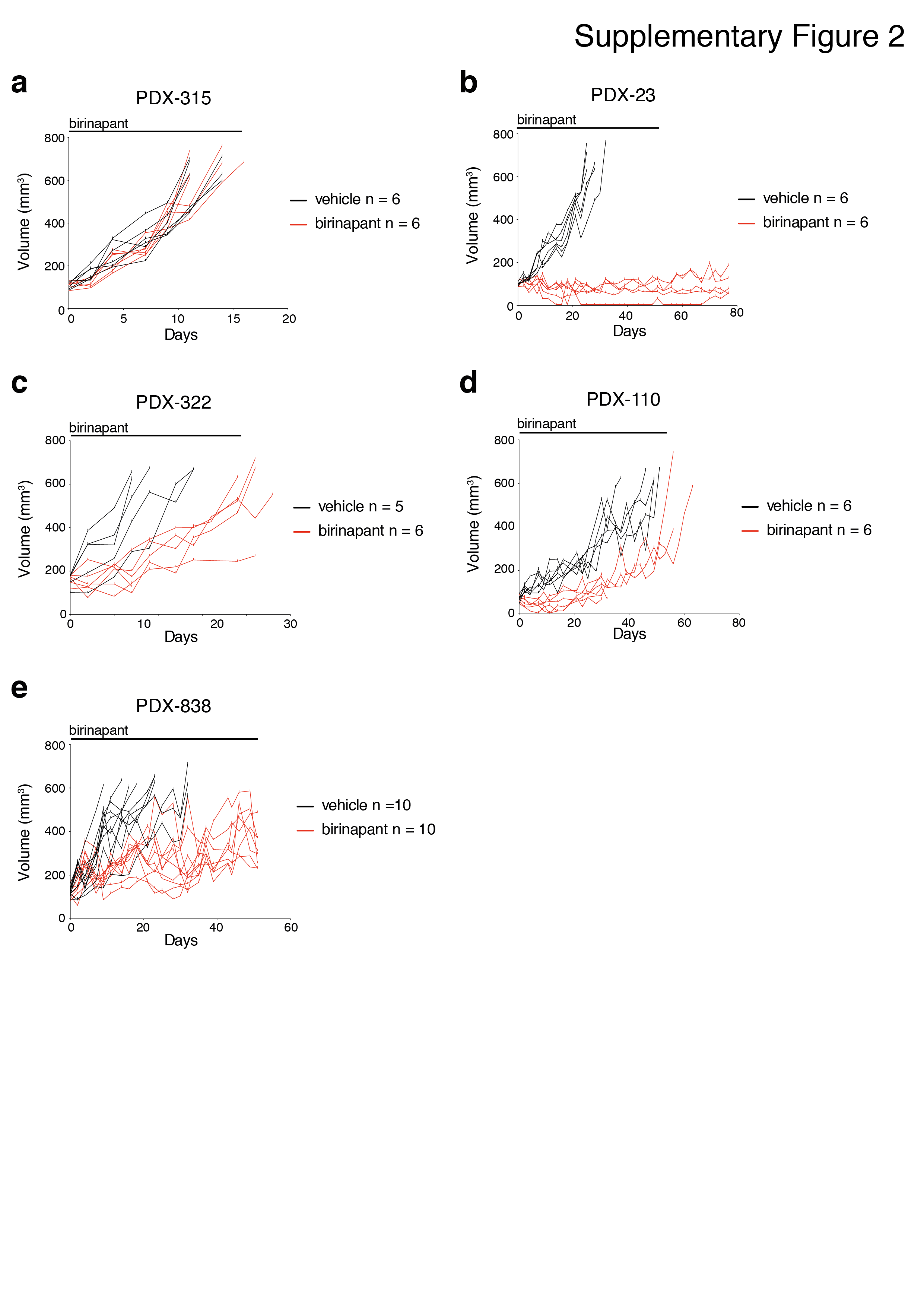

Supplement: Supplementary file 2 — SupFig2 [file 41418_2020_541_MOESM2_ESM.png]

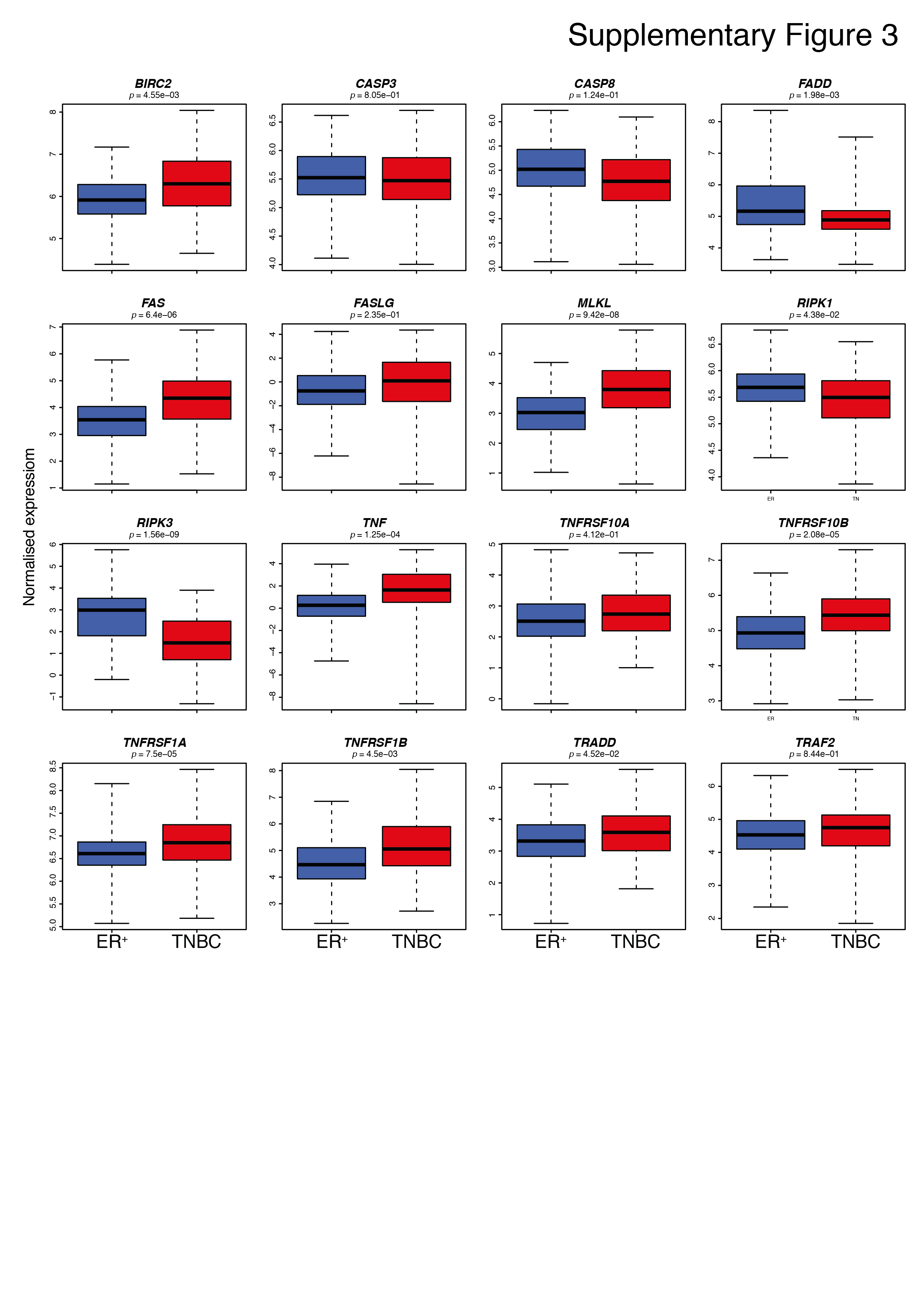

Supplement: Supplementary file 3 — SupFig3 [file 41418_2020_541_MOESM3_ESM.png]

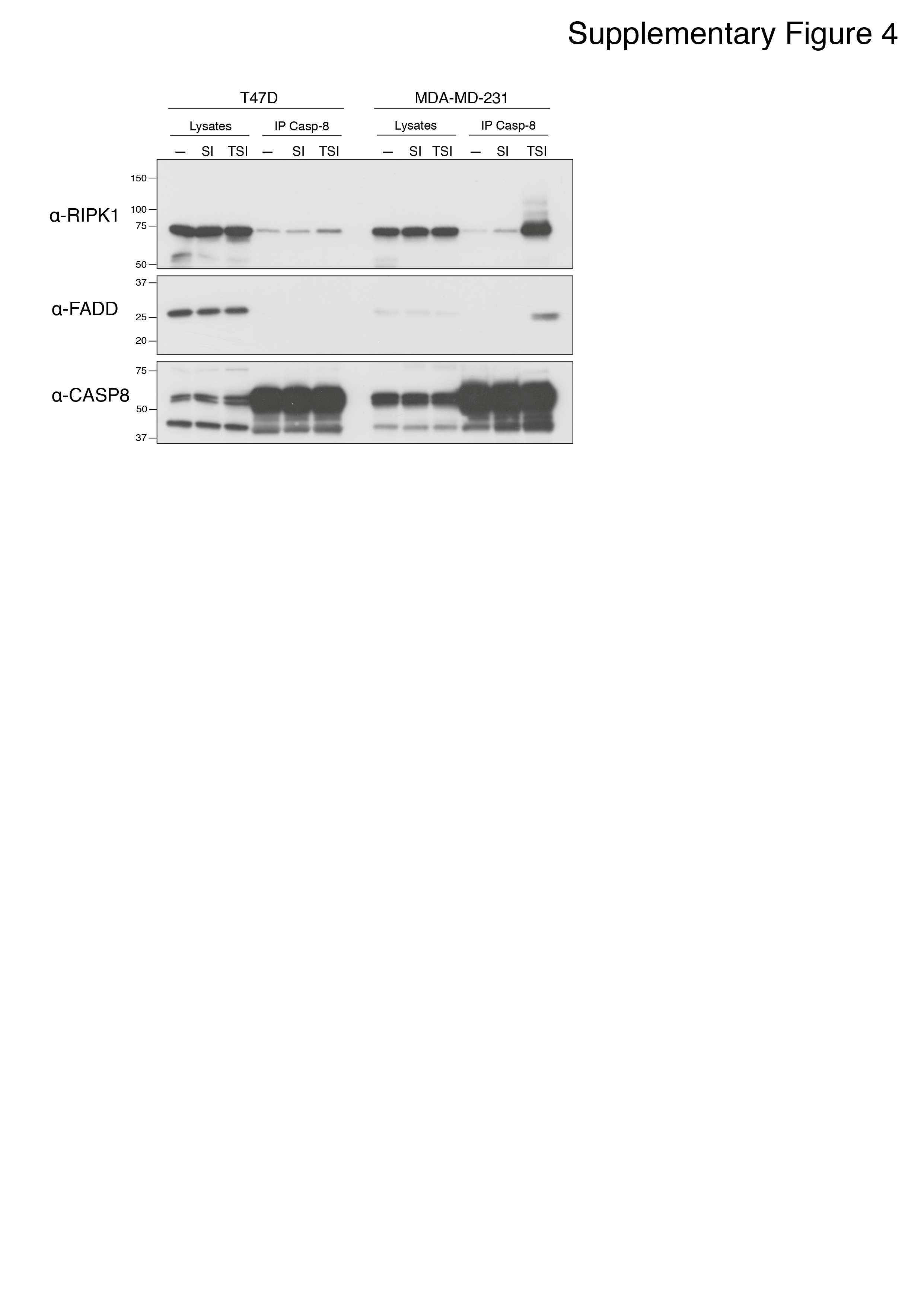

Supplement: Supplementary file 4 — SupFig4 [file 41418_2020_541_MOESM4_ESM.png]

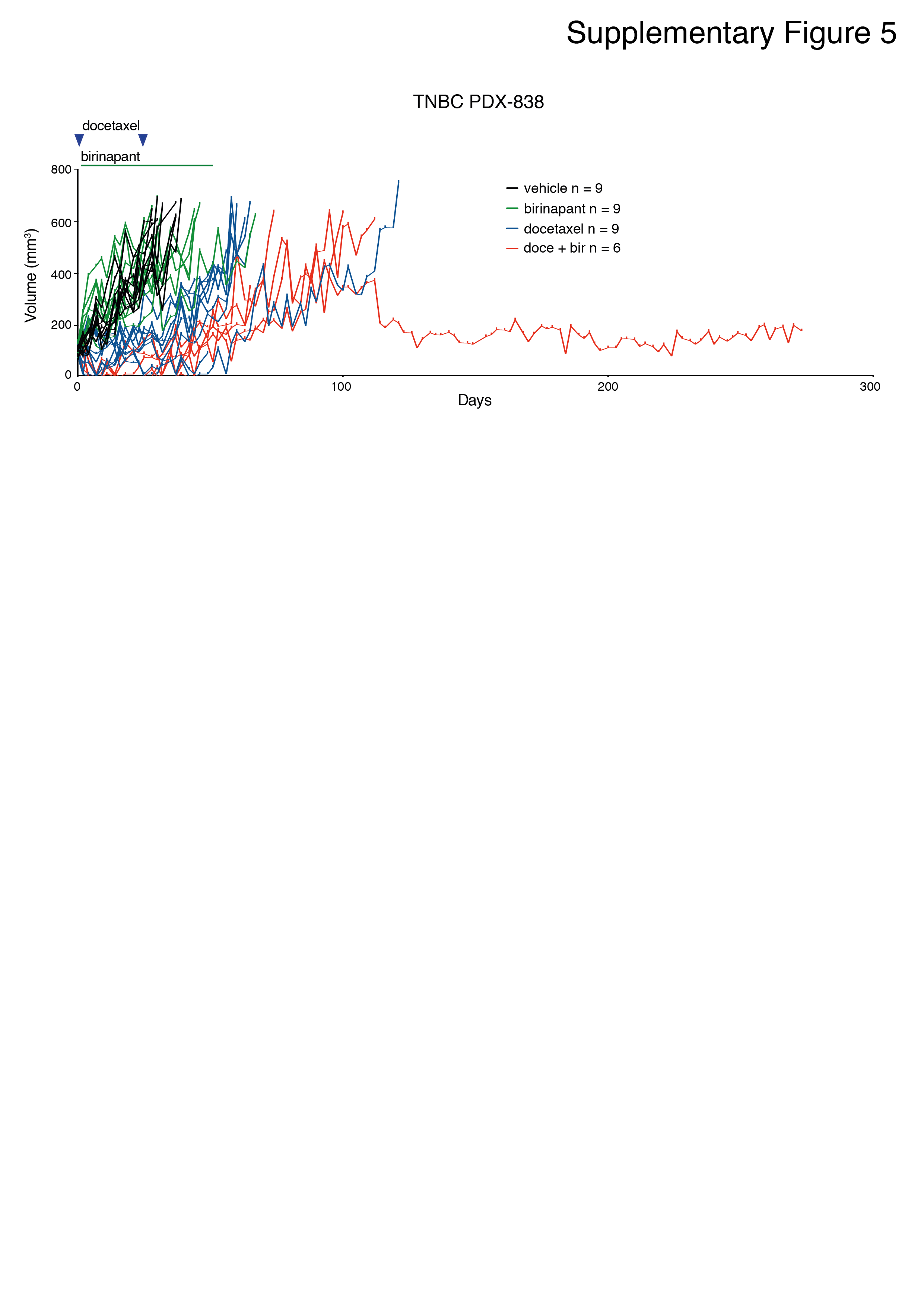

Supplement: Supplementary file 5 — SupFig5 [file 41418_2020_541_MOESM5_ESM.png]

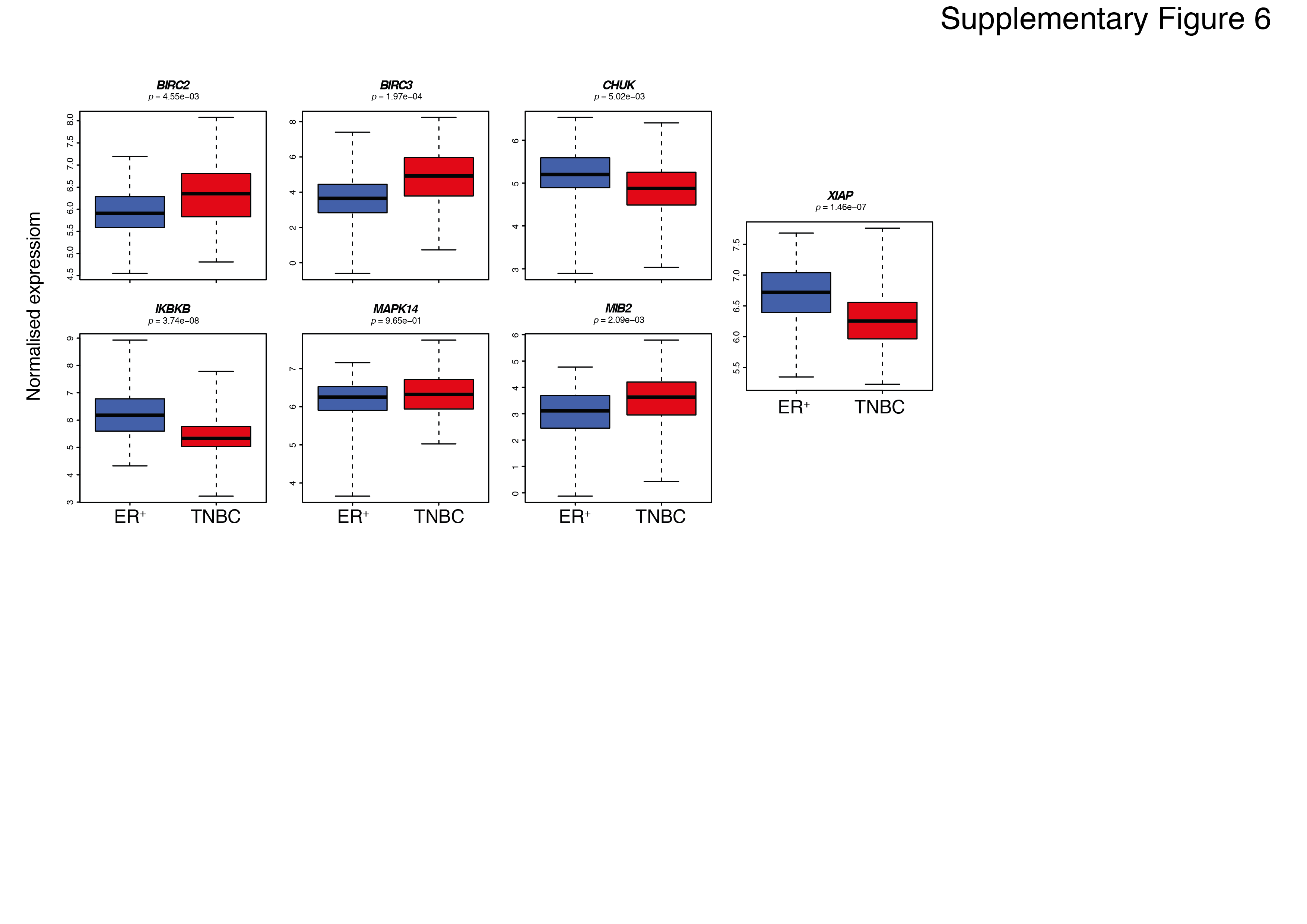

Supplement: Supplementary file 6 — SupFig6 [file 41418_2020_541_MOESM6_ESM.png]
