# Supplementary material for: Targeting triple-negative breast cancers with the Smac-mimetic birinapant
Source: Cell Death Differ. 2020 Apr 27;27(10):2768–80. doi: 10.1038/s41418-020-0541-0 (PMC7492458; doi:10.1038/s41418-020-0541-0)
Supplement: Supplementary file 7 — Supplementary Figures and Legends [file 41418_2020_541_MOESM7_ESM.docx]

**SUPPLEMENTARY FIGURES AND LEGENDS**

**Supplementary Figure 1: Safety of Birinapant *in vivo***

Blood counts at the time of euthanasia of mice treated intraperitoneally with vehicle or 30 mg/kg of birinapant for 8 weeks, 3 times/week. Graphs show mean ± SEM, n = 3 mice per group.

**Supplementary Figure 2: *In vivo* efficacy of birinapant in PDX models**

**A to E** Tumor volume curves for individual mice for the indicated PDX model (n = 6 to 10 mice per arm). Mice were treated with vehicle alone (black lines) or 30 mg/kg of birinapant (red lines), intraperitoneally 3 times/week. The treatment time is represented by the grey bars on top of the tumor volume curves. Mice were sacrificed when tumor size reached experimental ethical end point (> 600 mm^3^).

**Supplementary Figure 3: Expression of the ‘Smac-mimetic’ gene list in the TCGA database.**

Box plots representing the expression of indicated genes in TCGA samples (n = 132 for ER^+^ and n = 183 for TNBC samples).

**Supplementary Figure 4: Complex II analysis in breast cancer cell lines**

Western blot analysis of complex-II/Ripoptosome using anti-caspase-8 antibody. Cells were treated for 3 hours with either 1 μM of CompA (CpA) plus 5 μM of the caspase inhibitor IDN-6556 (SI) or with 100ng/ml of TNF plus 1 μM of CompA (CpA) plus 5 μM of the caspase inhibitor IDN-6556 (TSI). The caspase inhibitor IDN-6556 was used to stabilize the complex.

**Supplementary Figure 5: Birinapant sensitizes TNBC PDX tumors to conventional therapy *in vivo***

Tumor volume curves of individual mice for TNBC PDX-838 (n = 6 to 9 mice per arm). Mice were treated with vehicle alone (black line) or 15 mg/kg of birinapant alone (green line, intraperitoneally 3 times/week for 7 weeks) or with 10 mg/kg docetaxel alone (blue line, intraperitoneally on days 1 and 22) or with combined docetaxel and birinapant (doce + bir, red line). Mice were sacrificed when tumor size reached experimental ethical end point (>600 mm^3^).

**Supplementary Figure 6: Expression of other regulators of Smac-mimetic killing**

Box plots representing the expression of indicated genes in TCGA samples (n = 132 for ER^+^ and n = 183 for TNBC specimens).
